# Bacterial N_2_-fixation in mangrove ecosystems: insights from a diazotroph–mangrove interaction

**DOI:** 10.3389/fmicb.2015.00445

**Published:** 2015-05-11

**Authors:** Gabriela Alfaro-Espinoza, Matthias S. Ullrich

**Affiliations:** Molecular Life Science Research Center, Jacobs University BremenBremen, Germany

**Keywords:** nitrogen fixation, mangroves, diazotrophs, nitrogen cycle, bacteria–plant interaction, root colonization

## Abstract

Mangrove forests are highly productive ecosystems but represent low nutrient environments. Nitrogen availability is one of the main factors limiting mangrove growth. Diazotrophs have been identified as key organisms that provide nitrogen to these environments. N_2_-fixation by such organisms was found to be higher in the mangrove roots than in surrounding rhizosphere. Moreover, previous studies showed that mangroves grew better in the presence of N_2_-fixers indicating a potentially mutualistic relationship. However, the molecular signals and mechanisms that govern these interactions are still poorly understood. Here we present novel insights in the interaction of a diazotroph with a mangrove species to improve our understanding of the molecular and ecophysiological relationship between these two organisms under controlled conditions. Our results showed that *Marinobacterium mangrovicola* is a versatile organism capable of competing with other organisms to survive for long periods in mangrove soils. N_2_-fixation by this bacterium was up-regulated in the presence of mangrove roots, indicating a possible beneficial interaction. The increase in N_2_-fixation was limited to cells of the exponential growth phase suggesting that N_2_-fixation differs over the bacterial growth cycle. Bacterial transformants harboring a transcriptional *nifH*::*gusA* fusion showed that *M. mangrovicola* successfully colonized mangrove roots and simultaneously conducted N_2_-fixation. The colonization process was stimulated by the lack of an external carbon source suggesting a possible mutualistic relationship. *M. mangrovicola* represents an interesting genetically accessible diazotroph, which colonize mangrove roots and exhibit higher N_2_-fixation in the presence of mangrove roots. Consequently, we propose this microorganism as a tool to study molecular interactions between N_2_-fixers and mangrove plants and to better understand how changes in the environment could impact these important and relatively unknown interactions.

## Introduction

Mangrove ecosystems are wetlands located along tropical and subtropical coastlines. The term mangroves can also refer to a characteristic group of shrubs and woody tress that grow on brackish intertidal environments (Feller et al., 2010). These highly productive environments provide many ecosystem functions such as breeding and feeding grounds to diverse marine species, host a variety of organisms such as birds, mammals, and invertebrates (Holguin et al., 2001), acting as a barrier against tidal currents and tsunamis (Dahdouh-Guebas et al., 2005), and serve as carbon sinks, therefore, playing a key role in the global carbon cycle (Bouillon et al., 2008; Feller et al., 2010; Donato et al., 2011).

Although mangroves ecosystems are highly productive and rich in carbon, they are considered low nutrient environments. Nitrogen and phosphorus have been highlighted as nutrients limiting mangrove growth (Bashan and Holguin, 2002; Feller et al., 2002; Reef et al., 2010). Major factors contributing to nitrogen loss in these ecosystems are tidal export of nitrogen, denitrification, and the soil type (Boto and Robertson, 1990; Reef et al., 2010). In contrast, N_2_-fixation by diazotrophs is a major input of nitrogen to the ecosystem (Sengupta and Chaudhuri, 1991; Woitchik et al., 1997; Reef et al., 2010). N_2_-fixation in mangrove ecosystems has been detected in sediments, rhizosphere, decomposing leaves, tree bark, pneumatophores, and roots (Gotto and Taylor, 1976; Zuberer and Silver, 1978; Uchino et al., 1984; Hicks and Silvester, 1985; Holguin et al., 1992; Pelegrí et al., 1997). Moreover, N_2_-fixation rates were higher in mangrove roots as compared to the rhizosphere soil (Zuberer and Silver, 1978; Sengupta and Chaudhuri, 1991; Toledo et al., 1995; Ravikumar et al., 2004). Bashan et al. (1998) showed the transfer of fixed nitrogen by ^15^N experiments from N_2_-fixing bacteria to mangrove seedlings. Likewise, Ravikumar et al. (2004) found that several strains of diazotrophic *Azotobacter* increased mangrove root and shoot biomass, root length, and leaf area. These findings suggested a mutualistic interaction. Examples of N_2_-fixing microbes isolated from mangrove roots or sediments are *Marinobacterium mangrovicola*, *Listonella anguillarum*, *Vibrio campbelli*, *Azotobacter*, *Azospirillum*, and *Microcoleus* sp. (Sengupta and Chaudhuri, 1991; Holguin et al., 1992; Toledo et al., 1995; Ravikumar et al., 2004; Alfaro-Espinoza and Ullrich, 2014). In addition, phylogenetic studies on *nifH,* the gene encoding for the Fe protein of the nitrogenase enzyme complex and commonly used as a marker for N_2_-fixation (Zehr and McReynolds, 1989; Zehr et al., 2003) showed that members of the genera *Azotobacter*, *Derxia*, *Desulfuromonas*, *Sphingomonas*, *Pseudomonas*, and *Vibrio*, among others, could also play a role as N_2_-fixing organisms in mangrove ecosystems (Flores-Mireles et al., 2007; Zhang et al., 2008).

Although the description and isolation of microorganisms responsible for providing nitrogen to nitrogen-poor environments has been done extensively, very little advance has been made on understanding the effects that changing environmental conditions have on the cellular and ecological relationships between microbes and their mangrove environment. Previous studies indicated that some interactions between N_2_-fixers and mangrove plants are mutualistic (Bashan et al., 1998; Ravikumar et al., 2004). However, molecular signals and cellular mechanisms that govern these interactions are poorly understood. Postulating mechanisms similar to those extensively studied in legume crop plants might be impossible since the considered ecosystems and their contextual geobiochemical parameters differ significantly. Therefore, a genetically accessible N_2_-fixing bacterium and a mangrove plant species could strongly facilitate future studies and help us to better understand the importance of this potentially mutualistic interaction for the ecosystem and how future climatic changes could impact this organismal interplay.

Herein, we established the diazotroph *M. mangrovicola* Gal22 as an organism that interacts with the mangrove species *Rhizophora mangle*. For this we tested (a) the ecological competence and survival of *M. mangrovicola* in the mangrove’s rhizosphere and roots; (b) the influence of nutrient availability on the root colonization process; and (c) the genetic accessibility of the selected diazotroph. Corresponding results will provide the basis for further research on the symbiosis between diazotrophs and mangroves in these important tropical and subtropical ecosystems.

## Materials and Methods

### Mangrove Plants, Bacterial Strains, Plasmids, and Primers Used

Bacterial strains used in this study are listed in **Table 1**. All plasmids and primers used are listed in **Table 2**. *M. mangrovicola* Gal22 cells were cultivated overnight at 28∘C on HGB agar plates (supplemented with 0.1% yeast extract; Holguin et al., 1992). *Escherichia coli* DH5α cells were grown overnight at 37∘C on Luria–Bertani (LB) agar plates. *E. coli* ST18 was grown on LB agar plates containing 50 μg/ml 5-aminolevulinic acid. *M. mangrovicola* and *E. coli* transformants were maintained on HGB and LB medium supplemented with the appropriate antibiotic, respectively.

**Table 1:** Bacterial strains used.

Bacterial strains	Characteristic	Reference
***Escherichia coli***
DH5 ST18	supE44 D*lac*U169 (F80 *lacZ*DM15) *hsdR*17 *recA*1 *endA*1 *gyrA*96 *thi-*1 *relA*1 *pir* Δ*hemA pro thi hsdR*^+^ Tpr Smr chromosome::RP4-2 Tc::Mu-Kan::Tn7	Hanahan (1985) Thoma and Schobert (2009)
***Marinobacterium mangrovicola***
Gal22 Gal22.pBBR1MCS.GA.*nifH*::*gusA* Gal22ΔnifH Gal22ΔnifH.pBBR1MCS.GA.*gusA* Gal22ΔnifH.pBBR1MCS-3.GA.*gusA*	Wild type Wild type containing *nifH::gusA* fusion cloned in pBBR1MCS *nifH* deletion mutant, Cm^R^ *nifH* deletion mutant containing *gusA*, Cm^R^ *nifH* deletion mutant containing *gusA*, Tc^R^	Alfaro-Espinoza and Ullrich (2014) This study This study This study This study

*Cm, Chloramphenicol; Tc, tetracycline; ^R^, resistance*.

**Table 2:** Plasmids and primers used.

Plasmids/Primers	Characteristic	Reference
**Plasmids**		
pUC119	pMB1 origin, Ap^R^	Vieira and Messing (1991)
pSU23	p15A origin, Cm^R^	Bartolomé et al. (1991)
pKT210	IncQ, Cm^1^, Sm^R^	Bagdasarian et al. (1981)
pBBRlMCS	Broad-host-range cloning vector, Cm^R^	Kovach et al. (1994)
pBBRlMCS-3	Broad-host-range cloning vector, Tc^R^	Kovach et al. (1995)
pBluescript SK(+)	ColE1 origin, Ap^R^	Stratagene
pJET1.2	Replicon from pMBI vector, *eco47IR*, Ap^R^	Thermo Fisher Scientific
pJETnifHup/down	450-bp deletion of *nifH*. First and last part of *nifH* (fragment 1 and 2) cloned in pJET1.2	This study
pFCMl	Cm^R^ flanked by FRT sequences	Hoang et al. (1998)
pEX18TC	*oriT^+^ sacB^+^*, Tc^R^, MCS from pUC18	Hoang et al. (1998)
pEX.GA.nifH	Mutagenic construct. 2.7-kb HindIII fragment containing Cm^R^ flanked by the first and last part of *nifH* gene cloned in pEX18Tc	This study
pCAM140	Promoterless *gusA*, Ap^R^	Wilson et al. (1995)
pJETnif	1.5-kb fragment containing *nifH* plus upstream regulatory region	This study
pJETgus	1.9-kb fragment containing promoterless *gusA*	This study
pJETnif::gus	3.4-kb fragment containing *nifH::gusA* fusion	This study
pBBRlMCS.G *A.nifH::gusA*	3.4-kb HindIII fragment containing a transcriptional fusion of *nifH* gene plus upstream regulatory region with promoterless *gusA*. Cloned in opposite orientation with respect to *lac* promoter, Cm^R^	This study
pKW117	2,2-kb fragment containing *gusA* plus *tac* promoter and *trpA* transcriptional terminator, Ap^R^	Wilson et al. (1995)
pBBR 1MC S. G A.*gusA*	2,2-kb fragment containing *gusA* plus *tac* promoter cloned in pBBR1MCS, Cm^R^	This study
pBBRlMCS-3.GA.*gusA*	2.2-kb fragment containing *gusA* plus *tac* promoter cloned in pBBR1MCS-3, Tc^R^	This study
**Primers**	**Sequence (5′-3′)^∗^**	
Zf	TGYGAYCCNAARGCNGA	Zehr and McReynolds (1989)
Zr	ADNGCCATCATYTCNCC	Zehr and McReynolds (1989)
RTnifHf	ACCAGACTCGACGCACTTAA	This study
RTnifHr	CGAAGCTGGCAAGAAAGTCA	This study
RT16Sf	ACCACACACCTTTCCTCACA	This study
RT16Sr	CCAAGGCGACGATCTTTAGC	This study
nif-M-f	CCACTCGCCGGAGCATACGATGTA	This study
nif-M-r	ACACAGAACCTGGTAGCTGCCCTGG	This study
Fw.NotI.HindIII.f2	GCGGCCGCAAGCTTATCGGGCACTCGGACTGGAT	This study
Rv.BamHI.f2	GGATCCTACCGCGCTCTGGCACAGAA	This study
Fw.BamHI.fl	GGATCCCACCAGACTCGACGCACTT	This study
Rv.HindIII.fl	AAGCTTCCAGGATAAGGCTGTCGCT	This study
Mut.nifH.Fw	TGCCGACAACTTGCAATACC	This study
Mut.nifH.Rv	CCCAGAACACCATCATGGAA	This study
F.nifH.reg	AAGCTTCTGTTTTCCATACTCATGGCGG	This study
R.nifH.reg	GCATGCCGAATTACTCTTCAGCCGCAGTCT	This study
F.gus	GGCATGCTGATTGATTGA**AGGAGC**AACACT*ATG*TTACGTCCT GTAGAAAC^∗∗^	Egener et al. (1999)
R.gus	GTCTAGAAAGCTTAGTTGTTGATTCATTGTTTG	Egener et al. (1999)

*^∗^Underline are the restriction sites incorporated into the primer sequence: GCGGCCGC, NotI; AAGCTT, HindIII; GGATCC, BamHI; GCATGC, PaeI; TCTAGA, XbaI. ^∗∗^In bold, nifH RBS from Gal22; in italics, start codon. Ap, ampicillin; Cm, chloramphenicol; Tc, tetracycline; Sm, streptomycin; ^R^, resistance*.

*Rhizophora mangle* was selected as a host plant because it is a representative mangrove species that has a broad geographic distribution range, is easy to cultivate in the laboratory, and has seedlings which are commercially available in Europe.

### Isolation and Selection of the Bacterial Strain

N_2_-fixing bacteria were isolated and identified using the 16S rRNA gene as described in Alfaro-Espinoza and Ullrich (2014). The bacterial model strain was selected according to the following criteria: (a) the capability of the strain to fix N_2_, (b) its genetic accessibility and, (c) its ability to survive in *R. mangle* rhizosphere in a so-called fitness test when inoculated together with a group of other mangrove root-derived or sediment-borne indigenous bacterial strains.

N_2_-fixation was tested *in vitro* by the acetylene reduction assay (ARA) and confirmed by PCR amplification of the *nifH* gene with the degenerate primers Zr and *Z*f (Zehr and McReynolds, 1989; **Table 2**). Genetic accessibility was tested by transformation of bacterial strains using electroporation (Hamashitna et al., 1995; Wang and Griffiths, 2009) and conjugation, respectively. For this, the following vectors were used: pBBR1MCS (Kovach et al., 1994), pUC119 (Vieira and Messing, 1991), pSU23 (Bartolomé et al., 1991), and pKT210 (Bagdasarian et al., 1981; **Table 2**). The Fitness test was performed in an open water tank filled with artificial sea water [Tropic Marin (Dr. Biener GmbH, Wartenberg, Germany)]. *R. mangle* seedlings were cultured in pots filled with threefold autoclaved quartz sand (Carl Roth, Karlsruhe, Germany) mixed with 1/10 of sand from mangrove natural ecosystems [mangrove mud special (Marek Mangroven, Vienna, Austria)], and introduced into the sea water tank. After 6 months, the rhizosphere of the mangrove plants was inoculated with the isolated bacterial strains by mixing the sediment with bacterial inoculum without disturbing the seedling and its root system. The plants were kept at 25∘C with a photoperiod of 12 h. After 1 month of incubation, bacteria were re-isolated from the rhizosphere and mangrove roots by first (a) shaking the rhizosphere soil in liquid HGB medium for 30 min, and (b) macerating the mangrove roots. Then, the rhizosphere soil and homogenized roots were subjected to serial dilution and plated on HGB medium. Finally, single colonies were restreaked, and taxonomically identified by sequencing the 16S rRNA gene as described in Alfaro-Espinoza and Ullrich (2014). This experiment was repeated three times with two replicates per sample.

### Colonization of *R. mangle* Roots by *M. mangrovicola* Gal22

#### Generation of *M. mangrovicola* Mutant Gal22ΔnifH

All restriction enzymes used in this study were obtained from Thermo Fisher Scientific, Schwerte, Germany. A genomic DNA library was constructed to obtain the flanking regions of the *nifH* gene sequence from *M. mangrovicola* Gal22. For this, genomic DNA was treated with the endonuclease XhoI thereby generating DNA fragments of different sizes. The XhoI-treated DNA was randomly cloned in XhoI-treated pBluescript SK(+), and ligation products were introduced to *E. coli* DH5α. The following specific primers were used to pre-screen for the presence of the *nifH* gene of Gal22, nif-M-f, and nif-M-r (**Table 2**). A colony giving a PCR signal of 400-bp corresponding to the Gal22 *nifH* gene was selected. The plasmid of this transformant was isolated and its insert DNA was subsequently sequenced. A total sequence of 2.65-kb was obtained containing the 873-bp *nifH* gene and its flanking regions.

To develop a *nifH* gene deletion mutant, primers Fw.BamHI.f1 and Rv.HindIII.f1 (**Table 2**) were used to PCR amplify a 829-bp fragment (fragment 1) carrying the 5′-end and 558-bp of the upstream sequence of *nifH*. Primers Fw.NotI.HindIII.f2 and Rv.BamHI.f2 (**Table 2**) were used to amplify a 750-bp fragment (fragment 2) containing the 3′-end and a 597-bp downstream sequence of *nifH.* Next, both fragments were cloned separately in vector pJET1.2 (Thermo Fisher Scientific) resulting in plasmids pJETnifHup and pJETnifHdown, respectively. In a second cloning step, fragment 1 was cloned in pJETnifHdown after BamHI/XhoI treatment, resulting in pJETnifHup/down (**Table 2**). A 1.147-kb chloramphenicol resistance cassette (Cm^R^) was excised from pFCM1 (Hoang et al., 1998) and cloned in pJETnifHup/down with the help of a BamH1 treatment resulting in an insert fragment of 2.7-kb. Finally, the 2.7-kb fragment was cloned in vector pEX18Tc (Hoang et al., 1998) following a HindIII treatment. The resulting mutagenic construct was designated pEX.GA.nifH (**Table 2**). To obtain *M. mangrovicola* Gal22 deletion mutants, *E. coli* ST18 was heat shock transformed with pEX.GA.nifH and later used for biparental conjugation with *M. mangrovicola* Gal22.

Biparental conjugation was prepared by growing *M. mangrovicola* Gal22 overnight at 28∘C on HGB agar plates. *E. coli* ST18 containing the mutagenic construct was grown overnight at 37∘C on LB agar plates supplemented with 50 μg/ml 5-aminolevulinic acid (LB.AVA) and 25 μg/ml Cm. Bacterial cells were scratched from the plates thereby taking double amount of strain Gal22 as compared to strain ST18, resuspended in LB liquid media, and mixed with each other. The mixture was spotted on LB.AVA agar plates and incubated overnight at 28∘C. After this mating period cells were scratched from plates, resuspended in HGB media and for 15 min. The cell suspension was serially diluted and dilutions plated on HGB agar supplemented with 25 μg/ml Cm. Since strain ST18 cannot grow without the presence of AVA, only *M. mangrovicola* transformants containing the Cm^R^ from the mutagenic construct transferred by homologous recombination to the bacterial genome will be capable of growing on these agar plates.

Primers Mut.nifH.Fw and Mut.nifH.Rv (**Table 2**) were used to confirm the *nifH* gene knockout in *M. mangrovicola* Gal22. A PCR product of 740 bp was expected for wild type strain Gal22 while a corresponding 1,437-kb band was expected for mutant Gal22ΔnifH.

#### Transcriptional Fusion of *nifH* Gene to the β-Glucuronidase Reporter Gene (*nifH*::*gusA*)

A 1.510-kb fragment containing the *nifH* gene and a 632-bp sequence upstream of the transcriptional start site was amplified using primers F.nifH.reg and R.nifH.reg (**Table 2**). The promoterless β-glucuronidase (*gusA*) gene was amplified from plasmid pCAM140 (Wilson et al., 1995) with primers F.gus and R.gus (**Table 2**). Both PCR products were cloned individually in vector pJET1.2. The resulting plasmids were designated pJETnif and pJETgus, respectively. The insert DNA from pJETnif was cloned in pJETgus via PaeI/Xbal treatment thereby generating a *nifH*::*gusA* fusion in the designated pJETnif::gus (**Table 2**). The *nifH*::*gusA* fusion was excised from pJETnif::gus by HindIII treatment and cloned in vector pBBR1MCS. The orientation of the insert was chosen in such way that expression of the reporter gene by the vector-borne *lac* promoter is avoided. The obtained transcriptional fusion plasmid pBBR1MCS.GA.*nifH*::*gusA*, was introduced to *M. mangrovicola* by conjugation using a corresponding *E. coli* ST18 transformant.

In addition, the *gusA* reporter gene was excised from pKW117 (Wilson et al., 1995) by HindIII restriction and cloned in pBBR1MCS obtaining plasmid pBBR1MCS.GA.*gusA* (**Table 2**), which was introduced to Gal22 by conjugation using *E. coli* ST18. Additionally, *M. mangrovicola* Gal22ΔnifH was marked with another plasmid designated pBBR1MCS-3.GA.*gusA* (**Table 2**). This plasmid was generated by initially cloning the *gusA* gene from pKW117 via HindIII treatment in pBluescript SK(+), form which *gusA* was excised by XhoI/PstI treatment and cloned in pBBR1MCS-3 (Kovach et al., 1995).

#### Incubations of *M. mangrovicola* Gal22 and *M. mangrovicola* Gal22ΔnifH with *R. mangle* Roots

A basic HGB medium was modified to assess bacterial colonization on mangrove roots in form of a total of four variants (**Table 3**). *M. mangrovicola* carrying pBBR.GA.*nifH.gusA* was used in the two treatments with nitrogen-free HGB medium designated treatment -N/-C and -N/+C. In those cases, the *gusA* reporter gene will only be activated by the *nifH* promoter, consequently a blue color product will be generated when the β-glucuronidase interacts with the substrate 5-bromo-4-chloro-3-indolyl glucuronide (X-Gluc) (Carl Roth, Karlsruhe, Germany). Therefore, cells were only localized in places were strain Gal22 was actually fixing nitrogen.

**Table 3:** Description of the four different treatments used to assay colonization of *M. mangrovicola* Gal22 on *R. mangle* roots.

Treatment	Description	Strains and vectors	
		Gal22	Gal22ΔnifH
-N/-C	Lack of N and C sources	pBBR1MCS. GA. *nifH::gusA*	pBBR1MCS-3.GA.*gusA*
-N/+C	Lack of N source	pBBR1MCS. GA. *nifH::gusA*	pBBR1MCS-3.GA.*gusA*
+N^∗^/-C	Lack of C source	pBBR1MCS. GA.*gusA*	pBBR1MCS-3.GA.*gusA*
+N^∗^/+C	Supplemented with N and C sources	pBBR1MCS. *GA.gusA*	pBBR1MCS-3.GA.*gusA*

N, nitrogen; C, carbon. *NH_4_Cl was used as nitrogen source instead of yeast extract.

In treatments were nitrogen (0.1% NH_4_Cl) was supplemented to the medium (+N/-C and +N/+C), plasmid pBBR1MCS.GA.*gusA* was employed to localize *M. mangrovicola*. Since under these conditions strain Gal22 does not need to fix N_2_, the constitutively expressed *gusA* allowed to localize the cells. Similarly, pBBR1MCS-3.GA.*gusA* was used to mark *M. mangrovicola* mutant Gal22ΔnifH. Here, the expression of *gus*A reporter gene was driven by a *tac* constitutive promoter, which allowed us to localize the cells under any of the four treatment conditions.

The media used in all treatments was semi-solid with 0.3% agar content. 3-L glass beakers were filled with 500 ml of medium supplemented with 20 mg of X-Gluc and 12.5 mg of Cm. For treatments with *M. mangrovicola* Gal22ΔnifH 12.5 mg of Tc and 6.25 mg of Cm were added to 500 ml of medium. Subsequently, 6-months old *R. mangle* seedlings and bacterial suspensions (adjusted to obtain an initial OD_600_ of 0.07) were added to the beakers. The beakers were covered with aluminum foil to avoid light degradation of the X-Gluc substrate. Incubations were performed at room temperature (∼22∘C) for 20 days. The plants were exposed to a photoperiod of 12 h. During the incubation period, plants were visually examined for bacterial colonization and pictures were taken every 5 days. Three independent replicates per treatment were performed.

After the incubation period, *R. mangle* roots were removed, rinsed with phosphate buffered saline (PBS) and fixed overnight in 4% paraformaldehyde at 4∘C. After fixation, roots were kept in 70% ethanol for 5 min, then in 0.5 M sucrose in PBS solution for 1 h at 4∘C followed by an overnight incubation in 1 M sucrose in PBS solution at 4∘C. Roots parts showing bacterial colonization were embedded in Jung tissue freezing medium (Leica Microsystems, Nussloch, Germany) and stored at -20∘C. Subsequently, 15–20 μm transversal cuts were generated using a CM1900 cryomicrotome (Leica Microsystems). Finally, the roots were analyzed using an Axio light microscope (Carl Zeiss, Jena, Germany) and photographed.

### Quantification of *nifH* Gene Expression by Quantitative Reverse Transcriptase PCR

*Marinobacterium mangrovicola* Gal22 containing the vector pBBR1MCS was incubated in 60 ml of nitrogen-free liquid HGB medium (Holguin et al., 1992) supplemented with 25 μg/ml of Cm in a 1-L flask at 28∘C and shaking with 150 rpm and an initial optical density at 600 nm (OD_600_) of 0.07. The flask was sealed with a rubber septum and flushed with N_2_, and then the headspace was adjusted to 1% of oxygen. Same conditions were used for the incubation of *M. mangrovicola* Gal22 containing pBBR1MCS with 2 g of roots of *R. mangle*. After incubation, the cell cultures were harvested at an OD_600_ of 0.3, 0.5, and 0.7, respectively, using a protocol according to Schenk et al. (2008).

For RNA extraction the cell pellet was resuspended in RP buffer [3 mM EDTA, (0.5 M EDTA stock solution, pH 8), 700 mM NaCl, and DEPC H_2_O] with 40 mM DTT from a 1 M DTT stock solution added shortly before use. The cell suspension was added to pre-warmed lysis buffer [3 mM EDTA, (0.5 M EDTA stock solution, pH 8), 700 mM NaCl, 2% (w/v) SDS, and DEPC H_2_O], and shaken at 900 rpm for 3 min at 95∘C. A phenol/chloroform extraction followed as described by Schenk et al. (2008). After extraction, the RNA was treated with TURBO DNA-free Kit (Life technologies, Darmstadt, Germany) to remove contaminating DNA.

Absorbance at 260 nm (A_260_) was measured in a nanodrop 2000/2000c spectrophotometer (Thermo Fisher Scientific, Schwerte, Germany) to quantify the RNA concentration. The purity of the RNA was checked by determination of A_260_/A_230_ and A_260_/A_280_ ratios. Last, 500 ng of RNA were run on 1.5% agarose gel to determine its integrity. *nifH* gene expression was determined in a Eppendorf mastercycler ep realplex (Eppendorf, Wesseling–Berzdorf, Germany) using the QuantiTect SYBR Green PCR kit (Qiagen, Hilden, Germany). Amplification of the 16S rRNA gene was used as a reference. The following RT primers were used: RTnifHf and RTnifHr for *nifH* gene as well as RT16Sf and RT16Sr for 16S rRNA gene (**Table 2**). Relative quantification (ΔΔCq method) was used to analyze the *nifH* expression data. The complete experiment was performed three times with three independent replicates per sample.

## Results

### Isolation and Selection of the Bacterial Strain

Seven N_2_-fixing bacterial strains were isolated from the rhizosphere and roots of *R. mangle*. The isolated strains belong to the classes of Gammaproteobacteria and Alphaproteobacteria (Supplementary Figure S1). Our bacterial selection tests showed the following: there is a tendency that *in vitro* ARA rates of isolate Gal22 are higher than the corresponding rates of the other six isolates (Supplementary Figure S2). From the seven isolated bacterial strains used in the Fitness Test only three of the introduced bacterial isolates were recovered [isolate Gal22, isolate Gal12, and isolate Gal4 (Supplementary Table S1)]. Interestingly, only isolates Gal22 and Gal12 were recovered from all test samples and replicates. Finally, only isolates Gal22 and Gal12 exhibited DNA uptake using the transformation methods applied (Supplementary Figure S3). However, isolate Gal12 exhibit very low nitrogenase activity (Supplementary Figure S2), therefore, strain Gal22 was selected for further experiments because it best fulfilled our three selection criteria. Moreover, Isolate Gal22 was further characterized and taxonomically identified as *M. mangrovicola* Gal22 (Alfaro-Espinoza and Ullrich, 2014).

### Phenotypic Characteristics of *M. mangrovicola* Gal22 Transformants and Gal22ΔnifH Mutant

**FIGURE 1:**
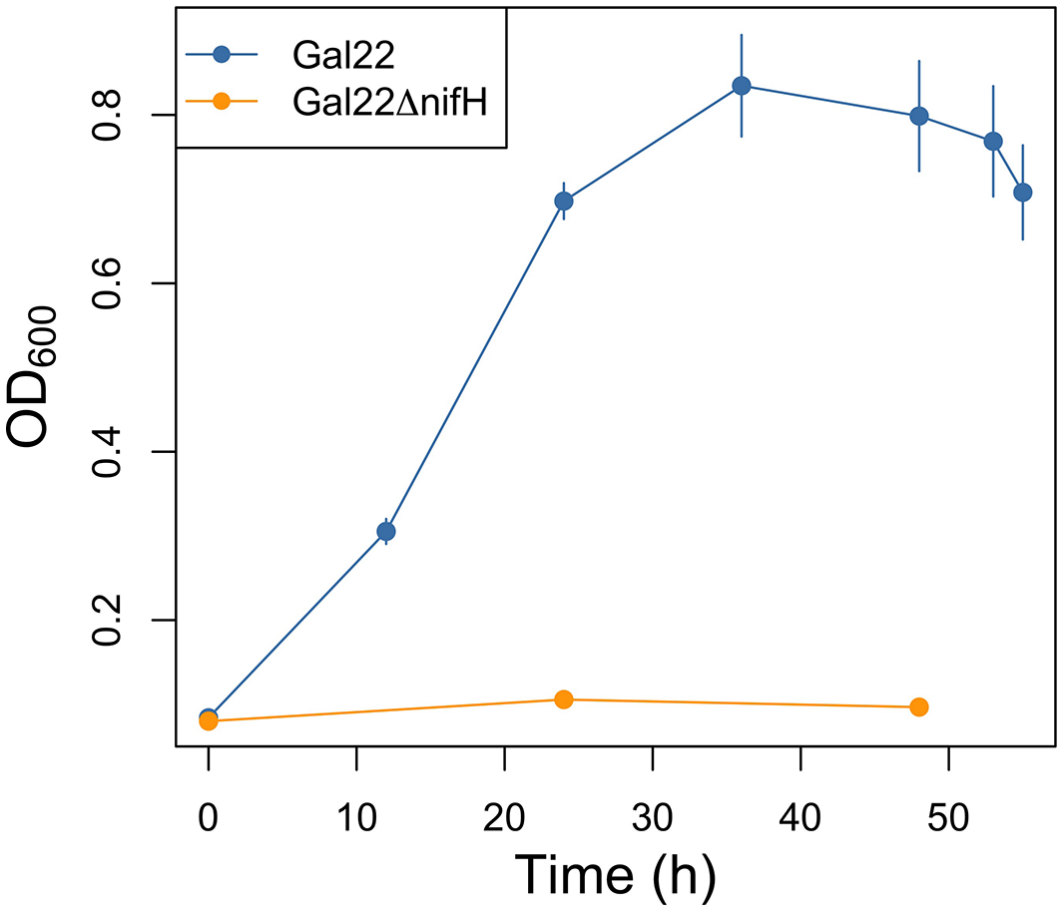
**Growth curve of Gal22 wild type and mutant Gal22ΔnifH in nitrogen-free HGB medium and 1% O_2_ in headspace flask**.

**FIGURE 2:**
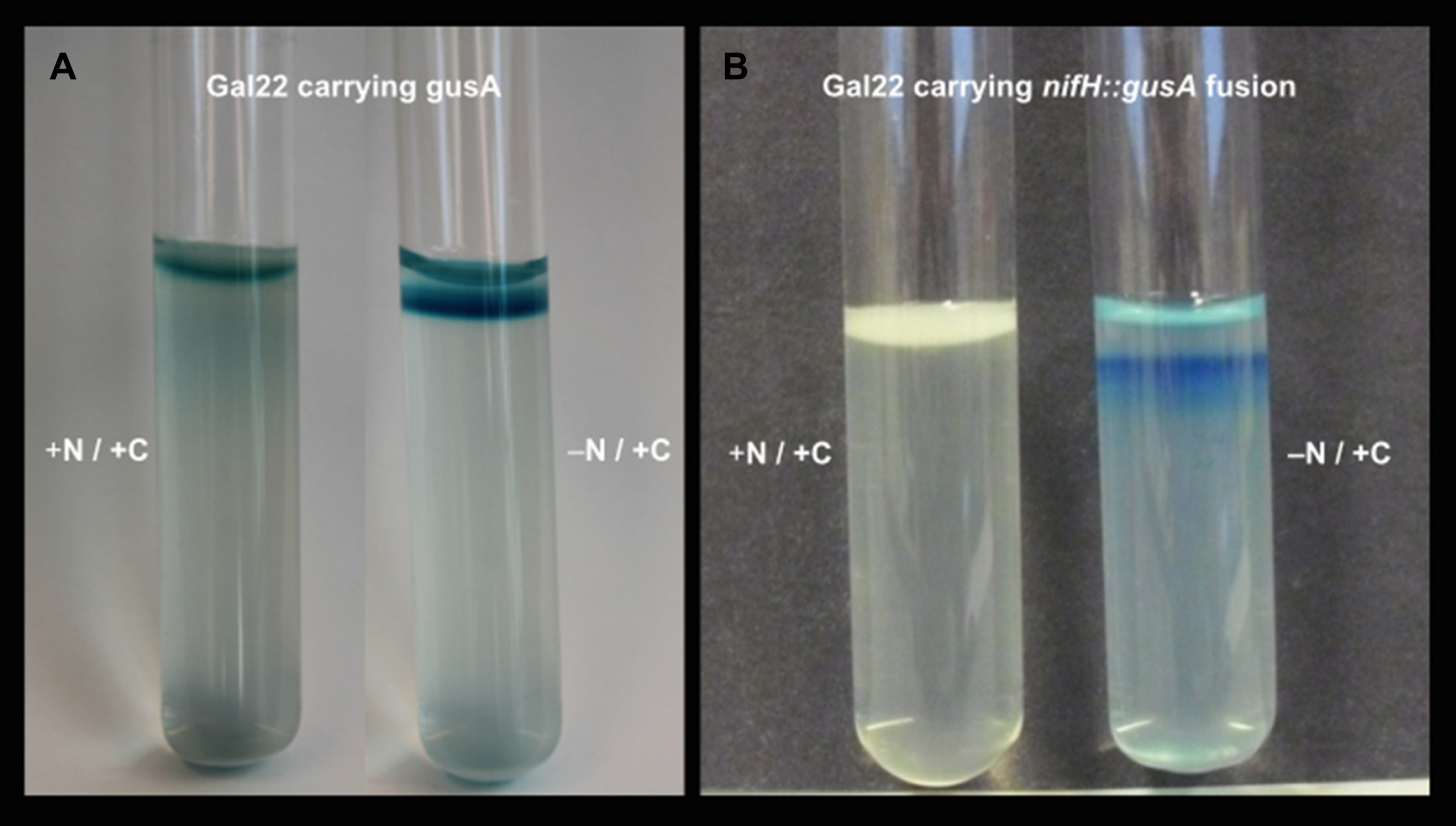
**(A)** *gusA*-carrying transformant of *Marinobacterium mangrovicola* Gal22*. gusA* expression is under the control of the constitutive promoter *tac*, and can be visualize in nitrogen-containing and nitrogen-free medium. **(B)** *M. mangrovicola* Gal22 transformant carrying the *nifH::gusA* fusion. *gusA* expression is under the control of the *nifH* promoter, and can only be visualized with a blue color formation under N_2_-fixing conditions in nitrogen-free medium.

Gal22ΔnifH mutant was genetically confirmed with a PCR product of 1,437-kb band, while a 740 bp was expected for wild type strain Gal22 (data not shown). Moreover its growth phenotype was visualized in liquid nitrogen-free HGB medium (**Figure 1**) showing that in contrast to the wild type the mutant is not capable of growing under N_2_-fixing conditions. **Figure 2** depicts the growth phenotype in semi-solid HGB medium of wild type transformants harboring reporter plasmids. Multiplication of bacterial cells carrying the constitutively expressed *gusA* reporter gene was observed in both, nitrogen-containing and nitrogen-free medium, respectively (**Figure 2A**). However, and as expected, when the *gusA* reporter gene was controlled by the *nifH* promoter, its expression could only be visualized when the cells were cultivated in nitrogen-free medium (**Figure 2B**).

### Colonization of *M. mangrovicola* Gal22 and its Gal22ΔnifH Mutant on *R. mangle* Roots

**FIGURE 3:**
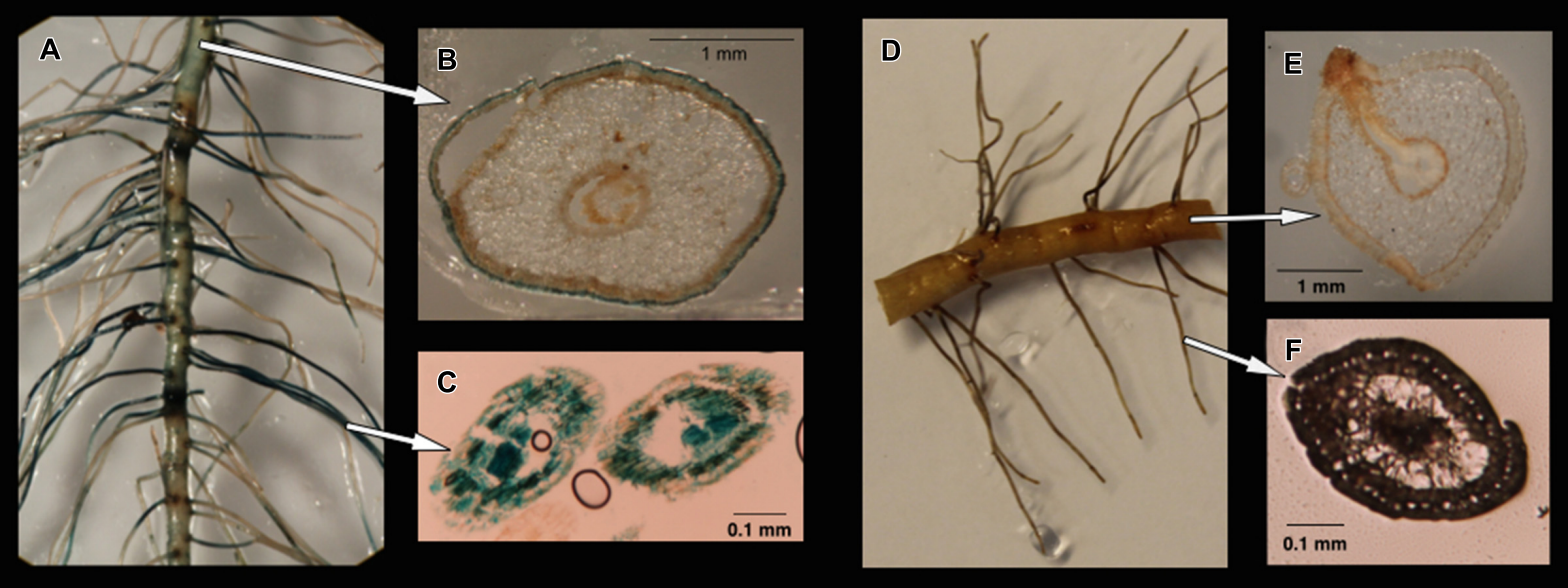
**(A)** Colonization of Gal22 transformant, carring a *nifH::gusA* fusion, on *Rhizophora mangle* roots after the treatment -N/-C. Cells are localized by the blue color formation from *gusA* activity. **(B)** Root surface bacterial colonization. **(C)** Colonization within young-fine lateral roots. **(D–F)** Root recovered after the treatment +N/+C. No bacterial colonization was observed in treatments were carbon and nitrogen were supplemented in the media.

Four treatments representing different nitrogen or carbon availabilities were applied to estimate the colonization patterns of *gusA*-carrying transformants of *M. mangrovicola* Gal22 and Gal22ΔnifH mutant on *R. mangle* roots. On lateral roots, colonization of Gal22 transformants was found on the surface and inside the finer lateral roots (**Figures 3A–C**). In treatments for which nitrogen and carbon were provided, *gusA* activity was observed on the soft-agar medium (data not shown) but bacterial colonization of roots or lateral roots was not visible (**Figures 3D–F**).

**FIGURE 4:**
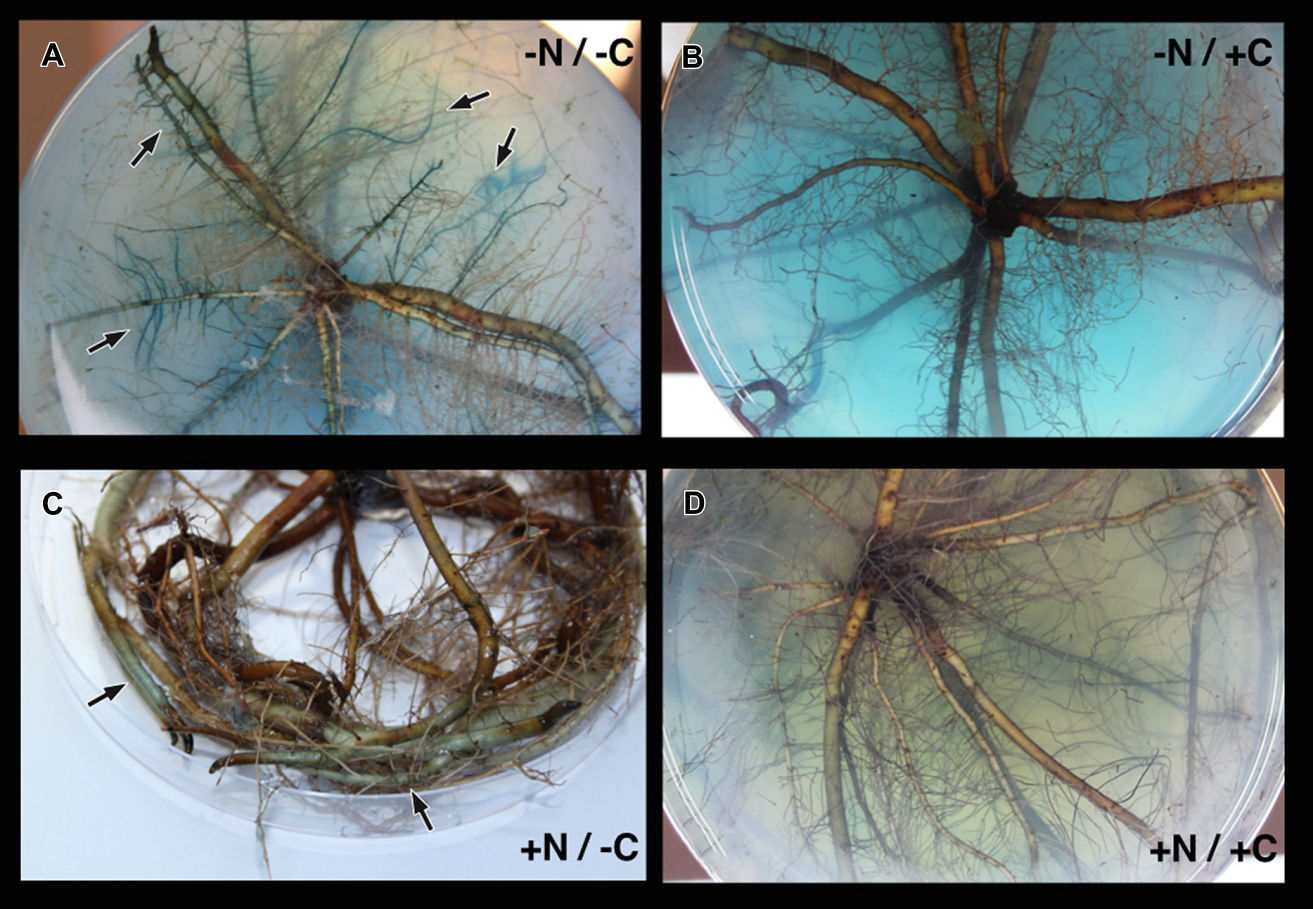
**Colonization of *M. mangrovicola* Gal22 transformants carrying *gusA* on *R. mangle* roots. (A)** Strong colonization and nitrogenase activity was visualized by the blue color formation from *gusA* activity in -N/-C treatment. In **(B,C)** there is a weak and moderate bacterial colonization, respectively. **(D)** Gal22 fail to colonize *R. mangle* roots when the surrounding media was supplemented with nitrogen and carbon.

**FIGURE 5:**
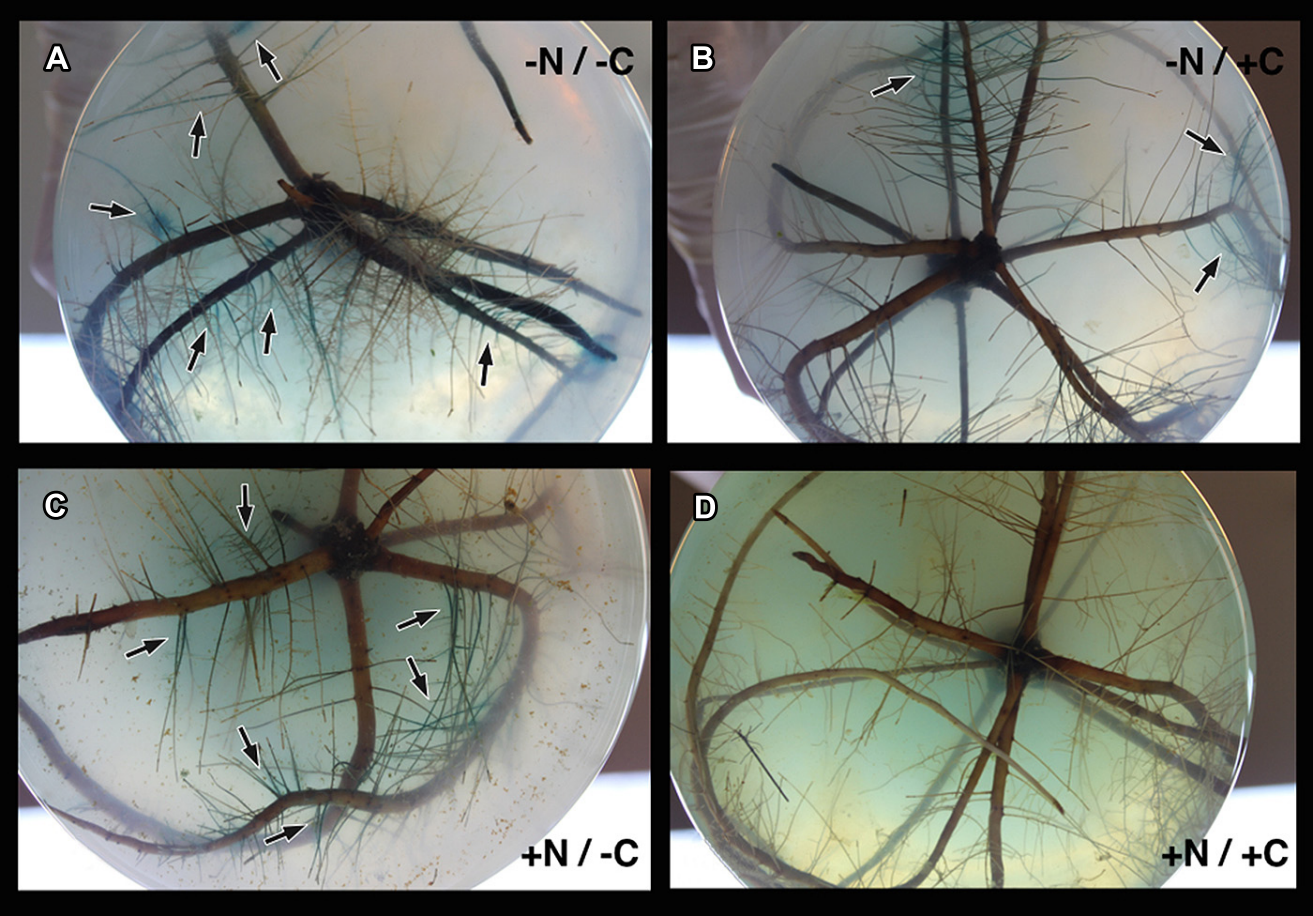
**Colonization of mutant Gal22ΔnifH on *R. mangle* roots. (A–C)** Strong colonization of Gal22ΔnifH under treatments lacking a source of nitrogen or carbon or both. **(D)** Gal22ΔnifH mutant root colonization was absent in treatments supplemented with nitrogen and carbon sources.

*Marinobacterium mangrovicola* Gal22 transformants carrying the *nifH*::*gusA* fusion exhibited a strong attraction to and a clear colonization of *R. mangle* roots when nitrogen and carbon sources were not provided in the semi-solid medium (**Table 4**; **Figure 4A**), when only carbon was lacking in the medium, the colonization was moderated (**Figure 4C**). Moreover, transcription of *nifH*::*gusA* revealed that the transformant used atmospheric nitrogen during root colonization under nitrogen and carbon limited conditions (**Figure 4A**). In contrast, when carbon or nitrogen or both were provided in the medium, the colonization efficiency of the Gal22 transformant with constitutively expressed *gusA* declined dramatically (**Figures 4B,D**). Interestingly, root colonization of Gal22ΔnifH transformants constitutively expressing *gusA* was strong in all treatments lacking either a nitrogen source, a carbon source or both suggesting that attraction to the roots was more pronounced when N_2_-fixation was abolished (**Table 4**; **Figures 5A–C**). In support of this, the mutant did not colonize mangrove roots when carbon and nitrogen sources were provided (**Figure 5D**).

**Table 4:** Colonization of *M. mangrovicola* Gal22 on *R. mangle* roots assayed under different treatments.

	Treatment			
	-N/-C	-N/+C	+N/-C	+N/+C
**Gal22**	Strong^ψ^	Weak^∗^	Moderate	Absent
**Gal22ΔnifH**	Strong^ψ^	Strong^ψ^	Strong^ψ^	Absent

*Less than 5%, mainly on root surface. ^ψ^More than 40%, mainly on fine lateral roots.

### Quantification of *nifH* Gene Expression by Quantitative Reverse Transcriptase PCR

**FIGURE 6:**
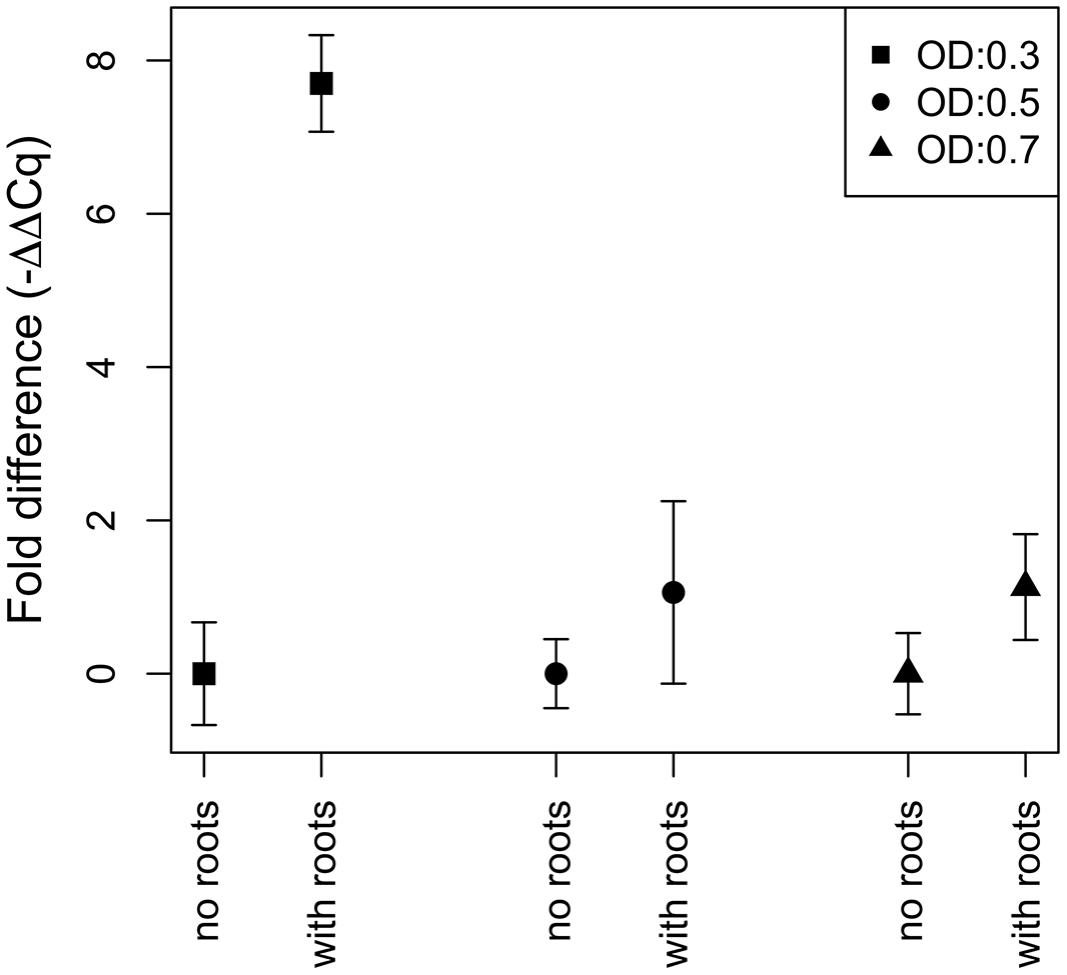
**Fold difference in expression of *nifH* after cells have been exposed to *R. mangle* roots**. The different symbols represent different treatments at optical densities (OD) of 0.3 (square), 0.5 (circle), and 0.7 (triangle). The only significant increase of *nifH* expression is during the exponential phase (OD: 0.3) at the presence of mangrove roots.

N_2_-fixation of strain Gal22 was studied by incubating the cells with or without presence of *R. mangle* roots (**Figure 6**). Transcription of *nifH* in Gal22 was significantly increased at low cell density (OD: 0.3; exponential phase) in the presence of the plant roots as compared to bacterial cells incubated without roots (**Figure 6**). However, during late exponential and stationary phases (OD: 0.5 and OD: 0.7) Gal22 did not show any significant mangrove root-mediated differences in *nifH* expression (**Figure 6**). Different growth phases of the wild type of Gal22 are depicted in **Figure 1** were an OD of 0.2–0.4 represents the exponential phase while an OD of 0.5–0.7 correspond to late exponential and stationary growth phases.

## Discussion

N_2_-fixing bacteria are common in mangrove ecosystems. Although various diazotrophs had been identified in both, the rhizosphere and the root, molecular mechanisms that control the interaction between N_2_-fixers and mangrove plants are not well understood. Herein we initially propose a genetically accessible diazotroph to study such interactions in order to foster our understanding of N_2_-fixation processes in mangrove ecosystems.

In previous studies, higher N_2_-fixation rates had been detected in mangrove roots as compared to the rhizosphere (Zuberer and Silver, 1978; Sengupta and Chaudhuri, 1991; Toledo et al., 1995; Ravikumar et al., 2004) suggesting that plants potentially obtain nitrogen through associated bacterial cells. Herein, it has been investigated if the presence of mangrove roots had an effect on N_2_-fixation of diazotroph Gal22. For this, we tested cells at different growth stages suggesting that mangrove root-induced expression of *nifH* differed remarkably at different bacterial growth rates. Interestingly, bacterial cells at late exponential or stationary phase did not showed significant mangrove root-inducible N_2_-fixation. Possibly, this might be due to the large amount of dead cells in these two growth phases since cellular content released by decaying cells could have provided sufficient amount of nitrogen to surviving bacteria making N_2_-fixation dispensable. Our results are consistent with those of a previous study conducted by Mulholland and Capone (2000), which showed higher N_2_-fixation rates at early exponential phase in *Trichodesmium* sp. and lower rates at stationary phase. Although, in general *M. mangrovicola* Gal22 showed comparable N_2_-fixation patterns to other microbes, this is the first time such a quantification have been made for mangrove associated diazotrophs.

One advantage of using a genetically modifiable organism such as *M. mangrovicola* Gal22 was the localization of bacterial cells inside of plant roots, on root surfaces, or in the surrounding semi-solid medium. The *gusA* reporter gene used in this study to localize bacterial cells on *R. mangle* roots showed that strain Gal22 colonized the surface of all roots and the interior of young lateral roots when no nitrogen or carbon were provided. Since tissue development of young lateral roots might not be completed yet, bacterial entry might have been facilitated. Similar results had previously been observed for a bacteria–rice interaction where the *gusA* system had been applied (Egener et al., 1999). To our knowledge, this is the first time that a reporter gene has been used to monitor colonization of mangrove roots by diazotrophs.

The colonization of *M. mangrovicola* Gal22 and Gal22ΔnifH mutant assayed under four different treatments revealed that the Gal22ΔnifH mutant was strongly attracted to the plant root possibly due to the lack of carbon and/or nitrogen in the surrounding medium. The lack of root colonization by Gal22 wild type and Gal22ΔnifH mutant when these nutrients were supplied confirmed these results. Since Gal22ΔnifH was not capable to obtain nitrogen by means of N_2_-fixation its attraction toward the roots was higher in comparison to that of wild type. The attraction of the Gal22 wild type toward the roots occurred mainly when a carbon source was missing suggesting that carbon availability on root surfaces might be a major colonization factor. Malic acid used as carbon source in this study had previously been shown to be present in root exudates (Jones, 1998; Kamilova et al., 2006; Rudrappa et al., 2008). Since a considerable amount of malic acid was provided in the treatment -N/+C, Gal22 did not approach roots to acquire it. Consequently and in support of this, very little attraction toward the roots has been seen when only nitrogen was lacking from the medium. Zuberer and Silver (1978) demonstrated that addition of a carbon source increased nitrogenase activity in sediments. Similar results were found by Flores-Mireles et al. (2007) and Zhang et al. (2008). In the -N/-C treatment the attraction of Gal22 toward the roots was strong and its *nifH* gene expression was high substantiating the above discussed results.

Since root exudates from *R. mangle* might attract Gal22 cells and foster high N_2_-fixation, our results may hint at a potentially mutualistic relationship between the diazotroph *M. mangrovicola* Gal22 and *R. mangle*. As showed by Ravikumar et al. (2004) some N_2_-fixers are beneficial for mangroves plants increasing root and shoot biomass and leaf area. *M. mangrovicola* Gal22 has proven to be a versatile organism being successful in competing and surviving for long periods on mangrove soil making it a good candidate for *in vitro* studies and potentially for field studies. Gal22’s N_2_-fixation is high when it colonizes mangrove roots, and the organism can be genetically manipulated. Due to this, we proposed *M. mangrovicola* Gal22 as organism to study molecular interactions between N_2_-fixers and mangrove plants. Further studies aiming to address the type of symbiotic relationship should focus on the transfer of the fixated nitrogen from Gal22 to the plant and on the molecular and cellular signaling of the bacterium during the interaction with mangrove roots. With the proposed organism a basis for future investigations has been established.

## Author Contributions

GA performed all experiments and wrote the manuscript. MU provided supervision and revised the manuscript. Both authors designed the study and interpreted the results.

## Conflict of Interest Statement

The authors declare that the research was conducted in the absence of any commercial or financial relationships that could be construed as a potential conflict of interest.
