# Supplementary material for: Bacterial N2-fixation in mangrove ecosystems: insights from a diazotroph–mangrove interaction
Source: Front Microbiol. 2015 May 11;6:445. doi: 10.3389/fmicb.2015.00445 (PMC4426756; doi:10.3389/fmicb.2015.00445)
Supplement: Supplementary file 1 [file Data_Sheet_1.PDF]

Supplementary Material

Bacterial N_2-fixation in mangrove ecosystems: insights from a diazotroph-mangrove interaction

Gabriela Alfaro-Espinoza[1]*, Matthias S. Ullrich[1]

[1]Molecular Life Science Research Center, Jacobs University Bremen, Bremen, Germany

*** Correspondence:** Gabriela Alfaro-Espinoza, Molecular Life Science Research Center, Jacobs University Bremen, Campus Ring 1, Bremen, 28759, Germany.
malfaroesp@jacobs-alumni.de

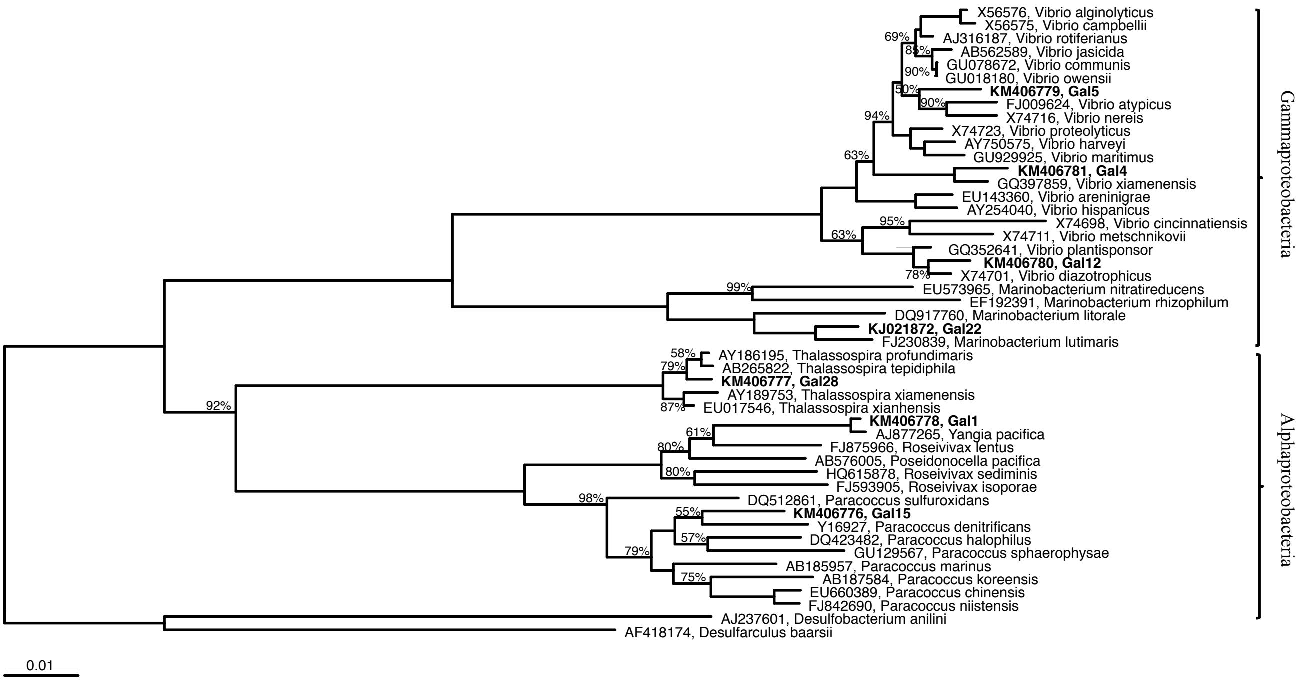

Supplementary Figure 1. Phylogenetic tree based on neighbor-joining method using 16S rRNA gene sequences. Bootstrap values of >50 % after 1.000 simulations are shown at the respective nodes. Bar, 0.01 substitutions per nucleotide position. Deltaproteobacteria sequences were used as outgroup.

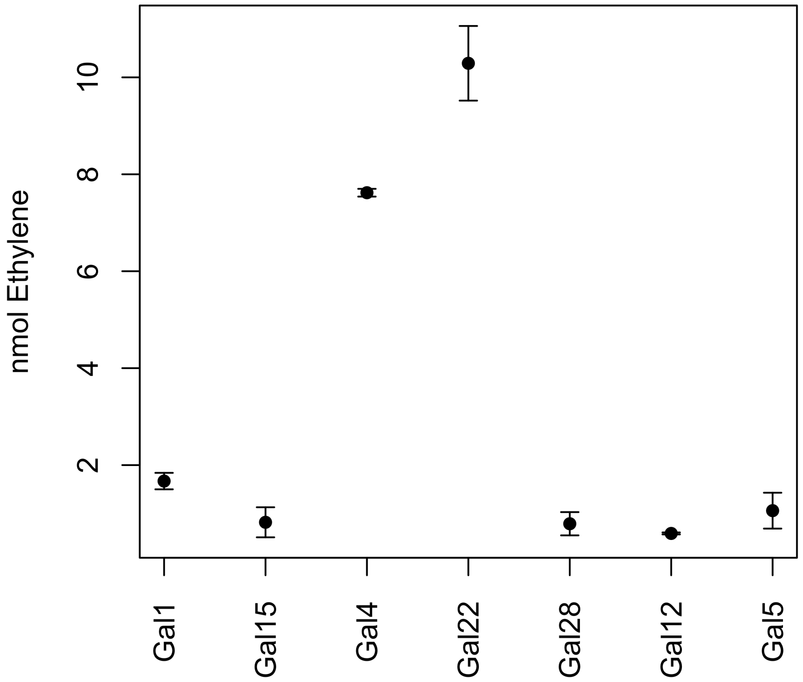

Supplementary Figure 2. Acetylene reduction assay of seven bacterial strains isolated from *Rhizophora mangle* roots.

Supplementary Table 1. Fitness Test: Organisms, isolates and sediment-born bacteria, recovered from mangrove roots and rhizosphere after one month of incubation.

**Recovered Organisms***	**Sample 1**	**Sample 2**	**Sample 3**	**Control** (No bacterial isolates added)
Isolate Gal12	+	+	+	−
Isolate Gal22	+	+	+	−
Isolate Gal4	−	+	−	−
Pseudoalteromonas sp.	−	−	−	+
Vibrio alginolyticus	+	+	−	+
Vibrio sp.	+	+	+	+
Bacillus sp.	−	−	−	+
Mangroveibacter sp.	−	+	+	+
Vibrio diazotrophicus	+	+	+	+
Zobellella denitrificans	−	−	−	+
Vibrio rhizosphaerae	−	−	+	−

+, organism found in the sample; **−**, organism absent in the sample
*16S rRNA sequences of recovered organisms were BLAST in NCBI database.

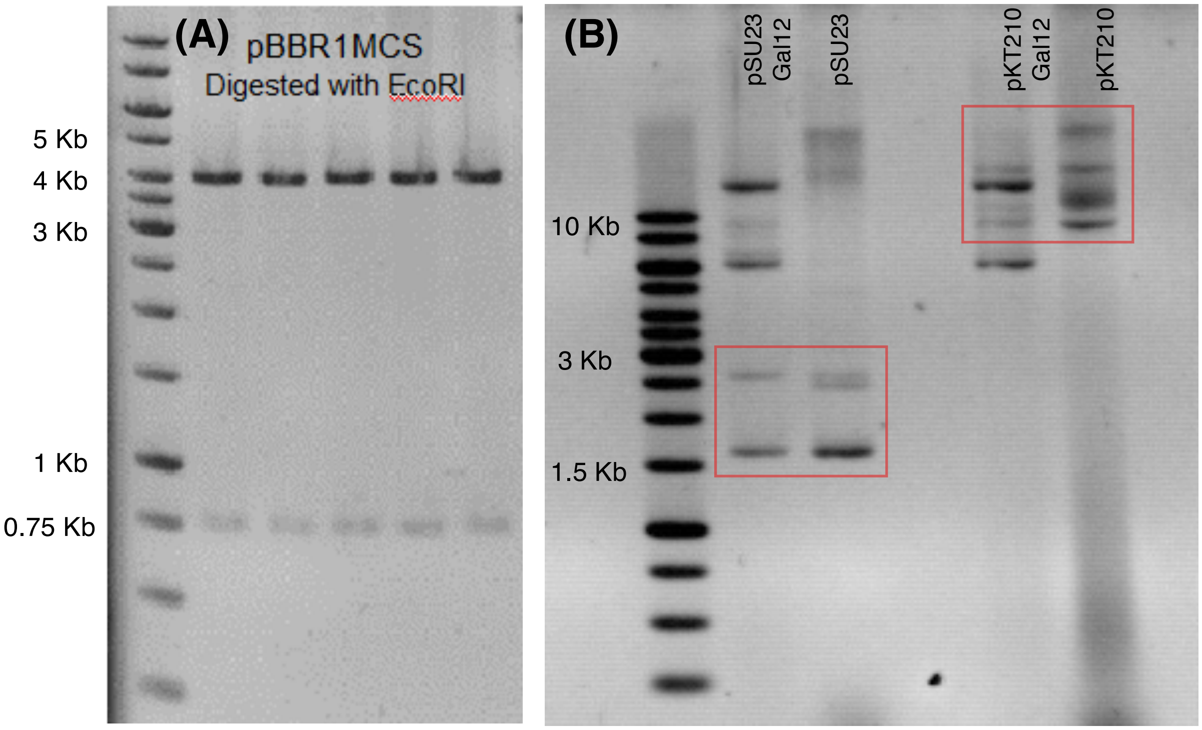

Supplementary Figure 3. (A) Electrophoresis gel showing plasmid pBBR1MCS after EcoRI treatment. pBBR1MCS was introduced in isolate Gal22 by conjugation. **(B)** Electrophoresis gel of isolate Gal12 transformed by electroporation with pSU23 and pKT210 plasmids. An undigested empty vector was run next to the undigested vector isolated from isolate Gal12. Bands within the red box correspond to the recombinant plasmid, while bands outside the red box correspond to the native plasmids.
